# NeuroCARE: A generic neuromorphic edge computing framework for healthcare applications

**DOI:** 10.3389/fnins.2023.1093865

**Published:** 2023-01-23

**Authors:** Fengshi Tian, Jie Yang, Shiqi Zhao, Mohamad Sawan

**Affiliations:** ^1^CenBRAIN Neurotech, School of Engineering, Westlake University, Hangzhou, Zhejiang, China; ^2^The Hong Kong University of Science and Technology (HKUST), New Territories, Hong Kong SAR, China

**Keywords:** epileptic seizure prediction, arrhythmia detection, hand gesture recognition, biomedical signal processing, neuromorphic computing

## Abstract

Highly accurate classification methods for multi-task biomedical signal processing are reported, including neural networks. However, reported works are computationally expensive and power-hungry. Such bottlenecks make it hard to deploy existing approaches on edge platforms such as mobile and wearable devices. Gaining motivation from the good performance and high energy-efficiency of spiking neural networks (SNNs), a generic neuromorphic framework for edge healthcare and biomedical applications are proposed and evaluated on various tasks, including electroencephalography (EEG) based epileptic seizure prediction, electrocardiography (ECG) based arrhythmia detection, and electromyography (EMG) based hand gesture recognition. This approach, NeuroCARE, uses a unique sparse spike encoder to generate spike sequences from raw biomedical signals and makes classifications using the spike-based computing engine that combines the advantages of both CNN and SNN. An adaptive weight mapping method specifically co-designed with the spike encoder can efficiently convert CNN to SNN without performance deterioration. The evaluation results show that the overall performance, including the classification accuracy, sensitivity and F1 score, achieve 92.7, 96.7, and 85.7% for seizure prediction, arrhythmia detection and hand gesture recognition, respectively. In comparison with CNN topologies, the computation complexity is reduced by over 80.7% while the energy consumption and area occupation are reduced by over 80% and over 64.8%, respectively, indicating that the proposed neuromorphic computing approach is energy and area efficient and of high precision, which paves the way for deployment at edge platforms.

## 1. Introduction

**FIGURE 1:**
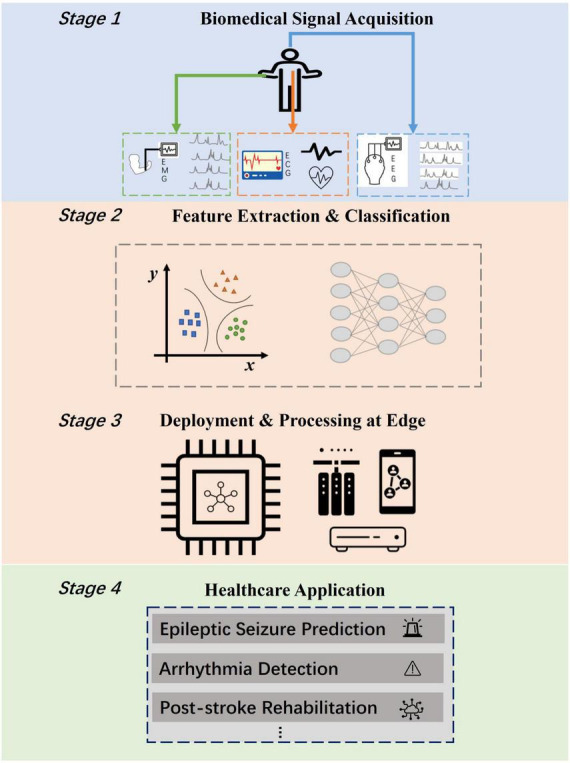
Edge computing scenarios for biomedical applications including four stages. The bottleneck lies in the realization of energy efficient feature extraction and classification approach (Stage 2) for deployment at edge (Stage 3).

The various biomedical datasets have provided the possibilities for the realization of a wide spectrum of medical applications. Electroencephalography (EEG) signals could be utilized for predicting epileptic seizure which is recognized as one of the most severe neurological diseases (Mirowski et al., 2009), motion recognition (Abiri et al., 2019), and stroke risk prediction which is ranked as the first leading causes of death and disability worldwide (Chen and Sawan, 2021). Electromyography (EMG) signals could pave the way for hand gesture recognition systems which have been proven to be immensely helpful in many human-computer interactive scenarios like treatment for post-stroke rehabilitation (Chen et al., 2019), and active prosthesis control (Parajuli et al., 2019). Electrocardiography (ECG) signals provide potentials for remote arrhythmia detection, which is one major cause of death nowadays according to World Health Organization (2017). The healthcare applications mentioned above always require at edge deployment for the convenience of patients and the enhancement of physician’s diagnosis. However, the computation and memory resources of edge devices are extremely limited. Figure 1 depicts at-edge system scenarios for various healthcare applications. Such systems consist of four common stages: biomedical signal acquisition stage, signal feature extraction and classification approach stage, edge platform deployment stage and application realization stage. To empower the realization of such systems, it is of great significance to propose a high-performance, energy-efficient, and generic feature extraction and classification approach for edge healthcare.

Recently, several AI-based algorithms have been used for various kinds of biomedical signals analysis tasks, like convolutional neural network (CNN) and recurrent neural network (RNN). A Short-Time-Fourier-Transform (STFT) based 2D-CNN was proposed for EEG signal processing by Truong et al. (2018a). Eberlein et al. (2018) designed a CNN with deep layers for seizure onset prediction on the time domain utilizing raw EEG signals. Xu et al. (2020) designed channel-wise convolution kernels to deal with seizure prediction and achieved a sensitivity of 98.2%. Atzori et al. (2016) and Wei et al. (2019) both applied CNN method to deal with EMG data. Hu et al. (2018) used a hybrid recurrent-convolution network to extract the sequential features of EMG signals, while Simão combined gated recurrent unit (GRU) and long-short term memory (LSTM) with the same aim (Simão et al., 2019). Kiranyaz et al. (2016) used 1D CNN to classify two major phases of ECG for arrhythmia detection and achieved sensitivity of 93.9%. The above methods all show advantages in classification accuracy. However, they all require tremendous amounts of computation resources, which proves to be a challenging task for edge platforms such as wearable and implantable devices. Recently, researchers have explored hardware friendly approaches for feature extraction and classification. Truong et al. (2018b) studied on deploying integer and binary weights in CNN, managing to achieve an over seven times reduction on weight size. Zhao et al. (2020) proposed a single dimension CNN with binary weights to deal with EEG data, which reduces the required memory for parameters by 86.12%. However, Truong et al. (2018b) still utilized integer weights of 4-bit width in their CNN and Zhao et al. (2020) still used fully precise values as dataflow in several layers of the neural network, which waste both energy consumption and computation resources. In conclusion, the existing approaches still face the drawback of balancing task performance with computation complexity. It remains as a problem to reduce data amount as much as possible while keeping the performance at a high level for edge biomedical applications.

Biologically inspired, spiking neural networks (SNNs), which stand as the most demonstrative form of neuromorphic computing approaches nowadays, have shown advantages in energy efficiency and performance in classification tasks (Kasabov, 2014; Srinivasan et al., 2016; Roy et al., 2019) and are expected to be the next generation of AI (Ghosh-dastidar and Adeli, 2009). To detect epilepsy seizure onsets based on EEG, Guo et al. (2017) designed an SNN utilizing supervised training methods and achieved an accuracy of 92.67%. Ma et al. (2020) designed an SNN based on reservoir computing for EMG classification. Amirshahi and Hashemi (2019) proposed an SNN for cardiac monitoring and achieved a sensitivity of 80.2%. Therefore, SNNs can provide a possible solution for deploying energy-efficient edge healthcare applications. Conventional unsupervised training methods like spike-timing-dependent plasticity (STDP) (Pu and Cook, 2015), Tempotron (Iyer and Chua, 2020), and SpikeProp (Shrestha and Song, 2018) are commonly utilized in existing SNNs (Pu and Cook, 2015; Amirshahi and Hashemi, 2019; Ma et al., 2020). However, it remains a problem to design and train spike-based neural networks as deep as CNN based on these methods (Tavanaei et al., 2019), which leads to limitations in both the performance and the applications of SNN. Although some work (Lee et al., 2018) try to improve STDP scheme for training multi-layer SNN, the performance is still insufficient to handle complicated signal analysis tasks. Combining the advantages of both CNN and SNN, Spiking CNNs are proposed to solve the drawbacks described above (Rueckauer et al., 2017). The weights of a Spiking-CNN are gained in CNN shadow training *via* backpropagation. The trained weights are then restored and correspondingly mapped onto the designed SNN. A unique spike encoder is required in spike-based computing methods to generate time-based spike sequences from the input data, which is specifically adapted to process various types of signals and tasks. In this way, Spiking-CNN succeeds to achieve high classification performance while much reduce the required computation resources compared to CNN. Diehl et al. (2015) did some primary work in the exploration of Spiking-CNN and conducted evaluation on MNIST dataset. Cao et al. (2015) also explored the potentials of Spiking-CNN in object recognition tasks. However, the effect of the spike encoder is overlooked in their work. For conversion based SNNs dealing with classification tasks, the key of keeping high performance is to rebuild the relative statistical data distribution of feature maps between layers in the format of spike sequences. The spike sequences are generated *via* spike encoder, which directly affects the data distribution of features.

To solve the existing drawbacks of unbalance among performance, versatility, and computation efficiency, we propose the implementation of NeuroCARE, a generic neuromorphic computing approach which is adaptive for various edge healthcare and biomedical applications. NeuroCARE first converts raw biologic signals to spikes through the proposed sparse Gaussian spike encoder, which rebuilds the relative statistical data distribution of feature maps in time domain. The dedicated channel-wise network structure of NeuroCARE manages to extract spatial-temporal features and stands as the foundation of accurate and efficient processing. The adaptive weight mapping scheme in NeuroCARE is codesigned with the spike encoder to achieve high performance. Moreover, NeuroCARE can be implemented on hardware in a totally multiplier free fashion which reduces both the hardware cost and power consumption significantly.

In our experiment, NeuroCARE reduces the computation complexity by over 80.7%, and the energy consumption and area occupation are reduced by over 80% and over 64.8%, respectively, showing advantages when compared to other systems with CNN topology.

The organization of this paper is as following. Section “2 Module and architecture designs” describes the proposed system architecture and design flow, where detailed modules of NeuroCARE are shown. Implementations, experiments, and relevant results are summarized in Section “3 Experiments and evaluation results,” respectively. Section “4 Conclusion” concludes this paper.

## 2. Module and architecture designs

**FIGURE 2:**
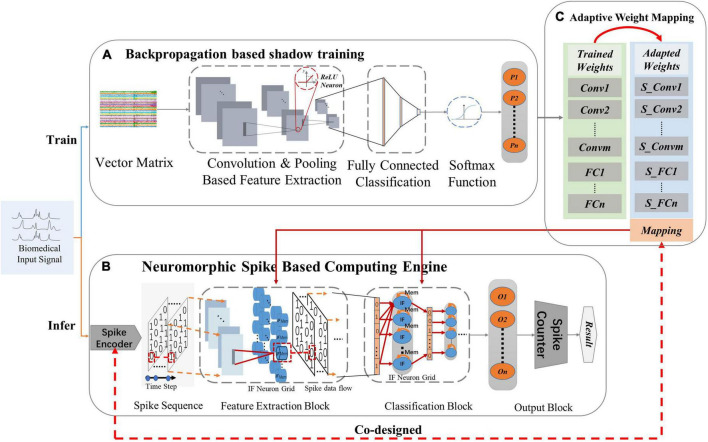
Processing strategies of the proposed NeuroCARE: **(A)** convolutional neural network (CNN) based processing for training, where “ReLU” stands for rectified linear activation function and “P” stands for the probability of one output result, **(B)** Neuromorphic spike-based computing engine for inferring, where “IF” stands for “integrate and fire”, “O” stands for “output neuron” and “n” stands for the number of output neurons, **(C)** Weight mapping, where “Conv” stands for “convolution layer”, “S_Conv” stands for “spiking convolution layer”, “m” stands for the number of “Conv”, “FC” stands for “fully connected layer” and “n” stands for the number of “FC”.

The processing strategy of NeuroCARE consists of two stages (Figure 2). The shadow training phase aims to get weights *via* backpropagation in the designed CNN topology (Figure 2A), which then transforms into neuromorphic SNN fashion (Figure 2B). The trained weights are first adapted and then mapped onto the Spiking-CNN *via* the proposed adaptive weight mapping method (Figure 2C) correspondingly. During the inference phase, a designed sparse Gaussian encoder is utilized to transform the biomedical signals to encoded spike sequences on the time domain. Then the neuromorphic engine operates inference to realize the classifications.

### 2.1. Sparse Gaussian spike encoder

In this study, we propose a sparse Gaussian spike encoding algorithm to generate time-dependent spike sequences with adaptive sparsity from the input continuous biomedical signals into and thus discrete the input data features in time domain.

Unlike other types of spike encoders such as (Donati et al., 2019) and (Davidson and Furber, 2021) where the encoder can only be applied to specific tasks, the proposed method can achieve better versatility and scalability with the codesign of the proposed weight mapping scheme. Such co-design scheme also ensures that the proposed method has the potential to apply on various network structure and various tasks. Relevant mathematical deduction will be presented in Part E of Section “2 Module and architecture designs.” Another priority of the proposed encoder lies in that it has better sparsity *via* mimicking the randomness of biological activities. Compared with prior rate-encoding and delta-encoding scheme, the proposed spike encoder could rebuild feature distributions in the time domain using far fewer time steps, thus bringing more efficiency. Moreover, the proposed spike encoder can be implemented in pure digital design and has good reconfigurability. Part F of Section “3 Experiments and evaluation results” presents the detail of circuit design of the spike encoder.

Algorithm 1 depicts the encoding process. The 2D input data sample is converted into a 3D vector with an extra time dimension. Meanwhile, key features of the original input are dispersed into multiple time steps after passing through the Gaussian spike encoder. The sparsity of the generated spike sequences is configurable utilizing several hyper-parameters: time-step, Vth-up and Vth-down. Time-step determines the length of time while Vth-up and Vth-down set a borderline for the generated Gaussian random values. The input original data for the spike encoder is raw biomedical sample. The encoded spikes are generated once at each time step *via* the proposed temporal Gaussian random discretization method. To determine elements of the encoded spikes, a Gaussian random matrix is generated at each time step. Holding the same shape as the original input sample, the generated matrix consists of random values following Gaussian distribution, whose variance is set as 1 and mean is set as (*V*_*th*_*up*_ + *V*_*th*_*down*_)/2. Elements of the generated random matrix is then one2one compared with the original input data. If the original element is greater, the corresponding encoded spike value is set one. Similarly, the spike value is set zero if the generated one is greater. This process applies to every time step, which means that the spike encoder generated one spike sample at each time step. If a vector in the shape of (C, H, W) (C, H, W stand for channel, height, and width respectively) is given as the input of the spike encoder, the output shall be (T, C, H, W) (T stands for time step) consisting of 0 or 1 elements.

**Algorithm 1:** Temporal Gaussian random sparse encoder

1 : Input: *time_step, original_data, V*_*th_up*_, V_*th_down*_ 2 : Output: *encoded spike sequences* 3 : for *t* = 1 : *time_step* do 4 : sample(t) ← Gaussian Random, mean = $\frac{V\,t\,h\,\_\,u\,p+V\,t\,h\,\_\,d\,o\,w\,n}{2}$ 5 : end for 6 : for *t* = 1 : time_step do 7 : for *data_value* in *original data* do 8 : if *sample*(*t*, data_*value*) < *original_data*(*datavalue*) 9 : *encoded_spikes*(*t*) ← 1 10: else 11: *encoded_spikes*(*t*) ← 0 12: end if 13: end for

The key benefits of spike encoder lie in that: (a) it discretized the information of raw biomedical data onto the time domain with adaptive sparsity, enabling the accurate and efficient Spiking-CNN processing method; (b) it helps to reduce the length of dataflow processed in the network from multi-bit (typically 8b/16b/32b) to merely 1-bit, and meanwhile still keeps the major features of input data so that the computation complexity and the required data memory is greatly reduced. The accumulation of the encoded spike sequences over all the time steps represents the retained information of input data. More data information is retained as the *time step* is set larger, which meanwhile brings more burden in memory and computing resources. In this work, different time steps are evaluated to compare the performance.

### 2.2. Channel-wise spatial-temporal network structure

For the processed raw biomedical signals, there always exist thousands or hundreds of elements in X axis (time axis) while only a few in Y axis (channel axis). Different channel records electrophysiological activities in different parts of human bodies. Thus, it may decrease the classification performance if the time (X) and channel (Y) axis are mixed up (Xu et al., 2020). Therefore, a channel-wise neural network structure is proposed in this work to avoid mixing up data features from different channels. The channel-wise neural network topology adopts single-dimension feature extraction kernels in convolution modules and max-pooling modules, which also brings convenience to edge hardware implementation.

As shown in Figure 2A, the structure of the CNN-based processing topology consists of three parts. The input vector matrices first go through the feature extraction part (FEP) which is composed of multiple convolution (Conv) layers and max-pooling (MP) layers. Rectified linear units (ReLU) are also connected after every feature extraction layer. The data after the feature extraction part is then sent to the classification part (CP) which is composed of fully connected (FC) layers. The outputs of FC layers are then processed in the SoftMax function part. The final outputs stand in the format of probability ranging in 0∼1 and the greatest one delivers the result of classification.

Figure 2B shows the structure of the proposed spike-based computing engine is shown in. Apart from the spike encoder and the spike counter, the proposed neuromorphic engine is mainly composed of a Spiking-CNN transformed from the CNN topology, which consists of a feature extraction block (FEB) and a classification block (CB). The FEB and CB have the same structure as FEP and CP of the CNN topology, respectively. The changes lie in that all the ReLU layers are replaced with IF neuron grids, which is explained detailly in Part C of Section “2 Module and architecture designs.” In Spiking-CNN, the data flow between two layers is transformed into the form of spikes (single-bit 0/1), which also transforms the “expensive” multiplication and accumulation (MAC) operations in CNN into the “cheap” adding-only (ADD) operations. Thus, Spiking-CNN requires far fewer computation resources than CNN, making it area-friendly and energy-efficient.

Table 1 shows the comparison of typical metrics between CNN, typical SNN, and this work. Since Spiking-CNN process data on time domain, the computing procedure can be executed both sequentially and parallelly, which depends on the architecture and resource constraints of the hardware processing element (PE), thus providing more flexibility for edge implementation.

**TABLE 1:** Overall comparison between convolutional neural network (CNN), spiking neural network (SNN), and this work.

	Comparison metrics	CNN	SNN	This work
Network structure	No. of FE Layer	*M_c_*	*M_s_*	$M_{s}^{\prime }$(= *M_c_*)
	Function layer	Softmax, ReLU	Delta/rate encoder, spike counter	Sparse Gaussian encoder, spike counter
	Input	2-D vector	Spike sequence (long time steps >100)	Spike sequence (few time steps ∼10/25)
	Output	Probability (multi-bit FP)	Number of spikes (INT)	Number of spikes (INT)
Neuron model	Activation function	ReLU function	LIF activation	IF activation
	Weight	*w*_*ij*_	*w*_*ij*_	$w_{i\,j}^{\prime }$ (Adaptive mapping)
	Bias	Vary between deff. layers	Vary between deff. layers	Constant △*V*
	Spike-based	–	✓	✓
	Dynamic leakage	Not required	Required	Not required
	FE domain	Spatial	Temporal	Spatial-temporal
Operation		Multiplication and accumulation (MAC)	Accumulation (ADD), dynamic leakage	Accumulation only (ADD)
Dataflow		Multi-bit value	Single-bit 0 or 1	Single-bit 0 or 1
Weight resolution		Multi-bit value		

### 2.3. Neuromorphic neuron model

**FIGURE 3:**
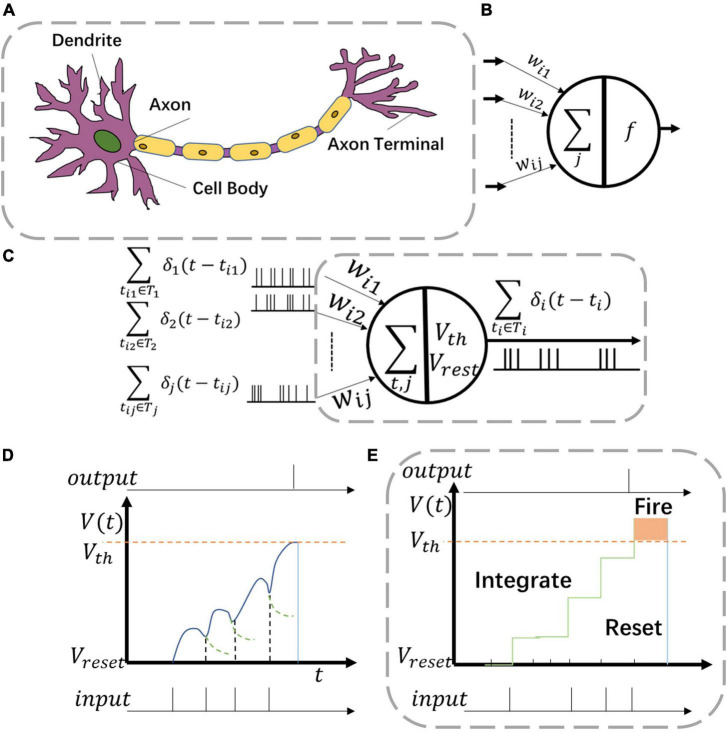
Various neuron models including **(A)** biological neuron, **(B)** artificial neuron, and **(C)** neuromorphic neuron. Two kinds of behaviors in panel **(C)** are **(D)** leaky-integrate-fire (LIF) function and **(E)** integrate and fire (IF) function.

In the proposed architecture, neuromorphic neuron grid is used as activation layer within each block (Figure 2B). As shown in Figure 3A, a biological neuron uses multiple dendrites to receive and send information. There exist one axon and many axon terminals at the end of the axon that connect with the dendrites of other neurons to transmit information. Neural models in NNs are designed in the purpose to mimic the function of biological neurons.

For common CNNs, the neuron model consists of inputs, outputs, and computation units (Figure 3B). The input connection is analogous to the dendrites of the neuron while the output is analogous to the axon. The computation units of CNN neurons complete the operations of multiplication and accumulation (MAC). However, such neurons are utilized only once in one single computation process.

For Spiking-CNNs, various biology-inspired neuron models have been explored (Roy et al., 2019). To mimic the behaviors of biological nerves, the neuromorphic neuron receives multiple spike sequences as inputs and may produce multiple output spikes as shown in Figure 3C. *V*(*t*) and *V*_*reset*_ stand for the values of membrane potential and resting potential of such neuron, respectively. When spike inputs arrive, *V*(*t*) increases, and later returns to *V*_*reset*_ after firing an output spike.

The leaky integrate and fire (LIF) neuron is a typical model to emulate biology neural behaviors. The behavior of LIF neurons is depicted in Figure 3D. However, the dynamic leaky process makes it hard to implement LIF neurons in high-speed digital circuits. In this study, the integrate and fire (IF) neuron, a simplified model with an adaptive threshold voltage *V*_*th*_, is utilized to propose a hardware-friendly approach. For spike based IF neurons, the membrane potential *V*_*i*_(*t*) is updated with time as shown in Eq. (1)


(1) $$\begin{array}{c} V_{i}\left(t\right)V_{i}\left(t-1\right)+Input\left(t\right)--Leakage \end{array}$$

In IF neurons, ∑δ_*j*_(*t*−*t*_*j*_) describes the sum of pre-synaptic stimulus of each neuron which also stands as the accumulation of spike inputs (Figure 3C) and leakage of IF neurons is set to be a constant voltage △*V* as show in Eq. (2)


(2) $$\begin{array}{c} V_{i}\left(t\right)V_{i}\left(t-1\right)+\sum_{j}wij\sum_{t\,j\in T}\delta j\left(t-tj\right)--△V \end{array}$$

As shown in Figure 3E, when the neuron membrane potential *V*_*i*_(*t*) exceeds the threshold voltage *V*_*th*_, a spike is fired at the output. Then *V*_*i*_(*t*) returns to the resting potential *V*_*reset*_.

### 2.4. Processing dataflow

To process raw biomedical data in the proposed neuromorphic engine, the original input data is first encoded into time-dependent spike sequences as described in Part A of Section “2 Module and architecture designs.” *Time Step* is first chosen. Other hyperparameters of spike encoder–*V*_*th_up*_ and *V*_*th_down*_ are set and adjusted along with the original input data distributions to achieve an optimal encoding performance.

At each point of time step, the encoded inputs are processed by the Spiking-CNN, where some of the output neurons may be activated and fire output spikes. The numbers of output spikes are recorded by the spike counter over all the time steps (Figure 2B) and the greatest one produces the result of classification.

In this study, the *V*_*reset*_ of all the IF neurons are set as 0 for the convenience to implement the whole neuromorphic engine on edge hardware. Threshold voltages *V*_*th*_ are set the same for each layer and adjusted along with the *V*_*th_up*_ and *V*_*th_down*_ to improve the performance.

### 2.5. Adaptive weight mapping configuration

As for the weight mapping which is shown in Figure 2C, the trained CNN weights are mapped on the Spiking-CNN in a way2way fashion after scaling with an adaptive scaling factor *f*.

Based on statical data distribution of activations in the proposed neural networks, we propose an adaptive weight mapping configuration method to keep high-performance classification for the conversion from CNN to Spiking-CNN. In the proposed mapping configuration, the scaling factor *f* is adaptive with the hyper parameters determined for the Gaussian spike encoder, i.e., *time*_*step*,*Vth*_*up*,*Vth*_*down*.

For the *Lth*-layer neural network, the weight vector connecting between the *k*_*th*_ neuron of layer *l-1* and the *i*_*th*_ neuron of layer *l* can be expressed as $W_{ik}^{l}$, *l* ∈ {1,…*L*}. For CNNs using ReLU neurons, the gained activation of neuron *i* in *l* layer is


(3) $$\begin{array}{c} a_{i}^{l}=M\,a\,x\,\left(0,\sum_{k}^{K^{l-1}}W_{i\,k}^{l}*a_{k}^{l-1}\right) \end{array}$$

where *K*^*l*−1^ represents the number of neurons in the *l-1* layer that are connected to the neuron *i* of *l* layer. Thus $a_{k}^{0}$ represents the *k*th value of the input data. Following the Gaussian distribution, the probability that the encoded spike value is 0 at the time step *t* is


(4) $$\begin{array}{c} P\left(S_{k\,t}^{0}=0\right)=\int_{V\,t\,h\,\_\,d\,o\,w\,n}^{a_{k}^{0}}\frac{1}{\sqrt{2\,\pi }}\exp\left(-\frac{\left(x-m\,e\,a\,n\right)}{2}\right)dx \end{array}$$

Similarly, the probability that the encoded spike value is 1 at the time step *t* is


(5) $$\begin{array}{c} P\left(S_{k\,t}^{0}=1\right)=\int_{a_{k}^{0}}^{V\,t\,h\,\_\,u\,p}\frac{1}{\sqrt{2\,\pi }}\exp\left(-\frac{\left(x-m\,e\,a\,n\right)}{2}\right)dx \end{array}$$

where


(6) $$\begin{array}{c} m\,e\,a\,n=\frac{V_{t\,h\,\_\,u\,p}+V_{t\,h\,\_\,d\,o\,w\,n}}{2} \end{array}$$

Therefore, the accumulated value of the encoded data $a_{k}^{0}$ through all the time steps is


(7) $$\begin{array}{c} Vac_{k}^{0}=\sum_{t}^{T}0*P\left(S_{k\,t}^{0}=0\right)+1*P\left(S_{k\,t}^{0}=1\right)\\ =T\,\int_{a_{k}^{0}}^{V\,t\,h\,\_\,u\,p}\frac{1}{\sqrt{2\,\pi }}\,e\,x\,p\,\left(-\frac{\left(x-m\,e\,a\,n\right)}{2}\right)\,dx \end{array}$$

Then we can get the scaling factor of the first layer


(8) $$\begin{array}{c} f_{1}=\frac{1}{K^{0}}\,\sum_{k}^{K_{0}}\frac{a_{k}^{0}}{V\,a\,c_{k}^{0}}=\frac{1}{K^{0}}\,\sum_{k}^{N}\frac{a_{k}^{0}}{T\,\int_{a_{k}^{0}}^{V\,t\,h\,\_\,u\,p}\frac{1}{\sqrt{2\,\pi }}\,e\,x\,p\,\left(-\frac{\left(x-m\,e\,a\,n\right)}{2}\right)\,dx} \end{array}$$

For the following layers of Spiking-CNN, considering the integrate-and-fire function of IF neurons, the accumulated value of the neuron *i* of *l* layer through all the time steps is


(9) $$\begin{array}{c} V\,a\,c_{i}^{l}=\sum_{t}^{T}\left(\sum_{k}^{K^{l-1}}f_{i}^{l}\,W_{i\,k}^{l}*S_{k\,t}^{l-1}-r^{l}*V_{t\,h}+V_{r\,e\,s\,e\,t}\right)\\ ≅T*\left(\sum_{k}^{K^{l-1}}f_{i}^{l}\,W_{i\,k}^{l}*r^{l}-r^{l}*V_{t\,h}+V_{r\,e\,s\,e\,t}\right) \end{array}$$

where $f_{i}^{l}$ stands for the scaling factor for neuron *i* in *l* layer and *r^l^* stands for the firing rate of the *l* layer which is defined as


(10) $$\begin{array}{c} r^{l}=0.5*\left(1-\frac{l}{L}\right) \end{array}$$

Combining Eq. (3), Eq. (9), and Eq. (10), we can get the scaling factor of the *l-th* layer in Eq. (11)


(11) $$\begin{array}{c} f_{l}=\frac{1}{K^{l-1}}\,\sum_{i}^{K^{l}}\frac{\frac{1}{T}*\sum_{k}^{K^{l-1}}W_{i\,k}^{l}*a_{k}^{l-1}+0.5*\left(1-\frac{l}{L}\right)*V_{t\,h}-V_{r\,e\,s\,e\,t}}{\sum_{k}^{K^{l-1}}W_{i\,k}^{l}*a_{k}^{l-1}} \end{array}$$

## 3. Experiments and evaluation results

Evaluations of NeuroCARE are conducted in terms of classification performance, energy consumption, and resource expenses in this study. We deploy this framework on a simulator based on Eyeriss architecture (Chen et al., 2017) and conduct three edge healthcare applications to demonstrate its advantages and versatilities: (1) epileptic seizure prediction based on EEG, (2) arrhythmia detection based on ECG, and (3) hand gesture classification based on EMG. Therefore, three different benchmark datasets are utilized to evaluate the performance of the proposed approach.

### 3.1. EEG based seizure prediction

The CHB-MIT EEG dataset is used to evaluate the performance of NeuroCARE on EEG-based seizure prediction tasks. The CHB-MIT dataset contains 23 measurements of scalp EEG data recorded from 22 patients at 256 Hz sampling rate (Shoeb, 2009). Among the total 23 measurements, 15 are recorded under the same fixed signal acquisition configuration using 23 electrodes, while the remaining 8 have some changes.

For seizure prediction tasks based on EEG signals, a tiny time window between the end of preictal interval and the beginning of seizure onset is defined as the seizure prediction horizon (SPH). The intervals chosen for the experiments are determined by SPH and the preictal interval length (PIL). In this experiment, we choose to use 30 min PIL and 5 min SPH. Using a fixed time window of 20 s, two categorized samples of the interictal intervals and the preictal intervals are extracted from the CHB-MIT dataset. For every extracted sample, the data height (H) is the number of recording channels, and the data width (W) is sampling-rate * 20 s, where each element is the recorded voltage value. Thus, the input vector shape (H, W) is 23 * 5,120 for this experiment. However, the interictal intervals contained in CHB-MIT dataset are much more than preictal intervals causing the problem of sample imbalance in training, which may lead to poor performances (Japkowicz and Stephen, 2002; Barandela et al., 2004). To overcome this barrier, samples of preictal intervals are extracted using 5 s overlapping (Tian et al., 2021).

In this study, only lead seizures occurring at least 4 h after the previous ones are taken into consideration (Hussein et al., 2019). Therefore, there are totally seven subjects suitable for the experiment. In this work, the results of evaluated metrics are the mean values of all the seven subjects.

With the ratio of 4:1, we separate the EEG dataset into training dataset and testing dataset randomly. The training dataset is used to gain the trained weights in CNN-based topology. The trained weights are first restored, then scaled, and finally mapped on the spike-based computing engine correspondingly. The performance of NeuroCARE is recorded on the testing dataset. When operating predictions, the raw EEG data is first transformed into spike sequences *via* the Gaussian spike encoder. The Spiking-CNN mapped with the scaled weights processes the encoded spike sequences and then produces results of prediction. Thus, the performance can be evaluated by comparing the results and the labels.

**FIGURE 4:**
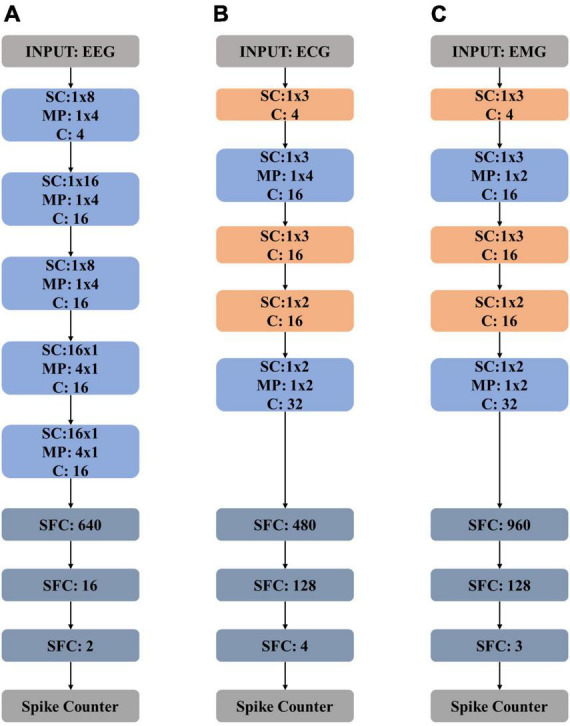
Model structures of **(A)** electroencephalography (EEG) network, **(B)** electrocardiography (ECG) network, and **(C)** electromyography (EMG) network. “SC” stands for “spiking convolution”, “MP” stands for “max pooling”, “SFC” stands for “spiking fully connected” and “C” stands for “channel”.

The neural network structure for EEG processing is detailed in Figure 4A. The proposed network is composed of five spiking convolution and max-pooling (SC and MP) layers, two spiking fully connected (SFC) layers, and two extra layers of spike encoder and spike counter.

### 3.2. ECG based arrhythmia detection

Evaluations on the performance of ECG-based arrhythmia detection tasks are conducted using MIT-BIH dataset in this work, which are obtained from total 47 subjects studied by the MIT-BIH Arrhythmia Laboratory. The MIT-BIH dataset contains forty-eight 30-min excerpts of two-channel ambulatory ECG recordings. Among the total 48 recordings, 23 are chosen randomly from 4k 24-h ambulatory ECG samples. These samples were recorded from a mixed collection of inpatients and outpatients with the ration of about 3:2 at Boston’s Beth Israel Hospital. The left 25 recordings include less common but clinically significant arrhythmia samples that cannot be well-represented in randomly chosen datasets, which are selected from the same set (Goldberger et al., 2000).

All the recordings are digitally sampled with a 11-bit resolution over a 10-mV range at 360 Hz per channel. As defined in standards from Association for the Advancement of Medical Instrumentation (AAMI), ECG recordings can be divided into five classes: ventricular ectopic beats (VEB), supraventricular ectopic beats (SVEB), fusion beats (F), non-ectopic beats (N), and unknown beats (Q). In total, MIT-BIH dataset contains 90,081 N samples, 7,008 VEB samples, 2,781 SVEB samples, 802 F samples, and 15 Q samples. In this experiment, to overcome the problem of data unbalance, we randomly selected 800 samples from N, VEB, SVEB, and F, respectively. Q samples are excluded in this experiment for there are too few to use for training. The total 3,200 samples are further separated into training dataset and testing dataset randomly in a 4:1 ratio.

The proposed ECG network structure is detailed in Figure 4B, which is composed of five spiking convolution (SC) layers, two max-pooling (MP) layers, and two spiking full-connected (SFC) layers. A spike encoder layer and a spike counter layer are also needed. The shadow weights of the CNN-based topology are first trained on the training data and then scaled and mapped on the spike-based computing engine correspondingly. The classification performance of the spike-based ECG network is then tested. The method of data processing in the inference procedure remains the same as the way mentioned in Part A of Section “3 Experiments and evaluation results.”

### 3.3. EMG based hand gesture recognition

To evaluate the performance on EMG based hand gesture recognition task, Nina Pro DB1 benchmark is used in this experiment. Nina Pro DB1 contains surface EMG (sEMG) data collection of 10 electrodes of measurements from 27 subjects (Atzori et al., 2014). All the data is collected with a sampling rate of 100 Hz. When collecting data, each type of hand gesture is repeated 10 times in a row. Every movement of hand gesture lasts for 5 s, and there exists a 3 s resting time between every two movements. Recordings of three kinds of commonly used hand gestures are utilized for evaluation of performance in this experiment, including the resting position, the thumb up position and the index flexion position. With the ratio of 4:1, the recorded samples are randomly separated into training dataset and testing dataset, respectively.

Input data is first encoded into spike sequences, and then processed by the proposed SNN. The size of the input sEMG samples during training and inference is 12 × 10 with a batch size of 32. Before converting into spike sequences in the time domain, the original samples are also min-max normalized into the range of (0,1).

Figure 4C shows the network structure. The proposed neural network consists of five spiking convolution (SC) layers, two max-pooling (MP) layers, and two spiking full-connected (SFC) layers with a spike encoder and a spike counter. In the training procedure, the training data is used to train the proposed CNN-based topology to get weights which are then scaled and mapped on the spike-based computing engine for inferring. The testing data is used to evaluate the performance of the spike-based engine.

### 3.4. Performance evaluations

In this study, to evaluate the classification performance of the proposed neuromorphic approach, we choose the standard evaluation metrics including accuracy, precision, recall, sensitivity, F1 score, and an overall score for the above-described multi-class (*N* classes) classification tasks, which are defined as follows:


(12) $$\begin{array}{c} A\,c\,c\,u\,r\,a\,c\,y\,\left(A\,c\,c\right)=\frac{\sum_{i=0}^{N-1}C\,l\,a\,s\,s_{i,c\,o\,r\,r\,e\,c\,t}}{\sum_{i=0}^{N-1}C\,l\,a\,s\,s_{i,t\,o\,t\,a\,l}} \end{array}$$

(13) $$\begin{array}{c} P\,r\,e\,c\,i\,s\,i\,o\,n=\frac{1}{N-1}\,\sum_{i=1}^{N-1}\frac{C\,l\,a\,s\,s_{i,c\,o\,r\,r\,e\,c\,t}}{C\,l\,a\,s\,s_{i,c\,r\,o\,r\,r\,e\,c\,t}+C\,l\,a\,s\,s_{0,f\,a\,l\,s\,e}} \end{array}$$

(14) $$\begin{array}{c} R\,e\,c\,a\,l\,l=\frac{1}{N-1}\,\sum_{i=1}^{N-1}\frac{C\,l\,a\,s\,s_{i,c\,o\,r\,r\,e\,c\,t}}{C\,l\,a\,s\,s_{i,c\,r\,o\,r\,r\,e\,c\,t}+C\,l\,a\,s\,s_{0,c\,o\,r\,r\,e\,c\,t}} \end{array}$$

(15) $$\begin{array}{c} S\,e\,n\,s\,i\,t\,i\,v\,i\,t\,y\,\left(S\,e\,n\right)\,\frac{C\,l\,a\,s\,s_{0,c\,o\,r\,r\,e\,c\,t}}{C\,l\,a\,s\,s_{0,t\,o\,t\,a\,l}} \end{array}$$

(16) F⁢1⁢S⁢c⁢o⁢r⁢e⁢ 2*P⁢r⁢e⁢c⁢i⁢s⁢i⁢o⁢n*R⁢e⁢c⁢a⁢l⁢lP⁢r⁢e⁢c⁢i⁢s⁢i⁢o⁢n+R⁢e⁢c⁢a⁢l⁢l

(17) $$\begin{array}{c} O\,v\,e\,r\,a\,l\,l\,S\,c\,o\,r\,e\,\frac{A\,c\,c+S\,e\,n+F\,1\,S\,c\,o\,r\,e}{3} \end{array}$$

where *Class*_0,total_ stands for the number of the normal state or the resting state and *Class*_*i,total*_ stands for the number of the *i*_*th*_ class of the processed biomedical data.

**FIGURE 5:**
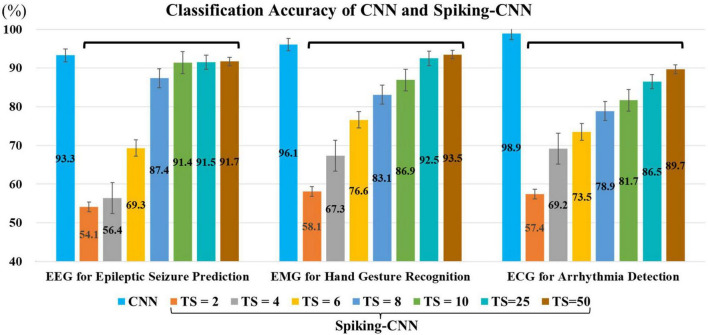
Classification accuracies for electroencephalography (EEG), electromyography (EMG), and electrocardiography (ECG) utilizing convolutional neural network (CNN) and Spiking-CNN with different configurations in time step, where “TS” stands for “the number of time steps” for Spiking-CNN. The larger TS is set, the higher accuracies are gained.

The effect of the set number of *time step* (TS) of the proposed spike encoder is shown in Figure 5, where the larger TS is, the higher accuracy is gained.

For the evaluation of computation efficiency of the proposed approach, the number of computing operations (ADD and MUL) and the required weight memory are estimated. The metric of *computation complexity* (TC) is also calculated to quantize the reduction of computation costs brought by the proposed approach. The calculation of TC of CNN and the proposed Spiking-CNN are presented in Eq. (18) and Eq. (19), respectively


(18) $$\begin{array}{c} TC_{C\,N\,N}=\sum_{a\,l\,l\,l\,a\,y\,e\,r\,s}M_{H}M_{W}\left(K_{H}K_{W}+K_{H}+K_{W}-1\right)C_{i\,n}C_{o\,u\,t}\times \\ O\,p\,s\times b\,i\,t+\sum_{a\,l\,l\,l\,a\,y\,e\,r\,s}N_{i\,n}\,N_{o\,u\,t}\times O\,p\,s\times b\,i\,t \end{array}$$

(19) $$\begin{array}{l} TC_{SCNN}=\sum_{all\;layers}M_{H}M_{W}\left(K_{H}+K_{W}-1\right)C_{in}C_{out}\times \\ \;Ops\times bit\times TS+\sum_{all\;layers}N_{in}N_{out}\times Ops\times bit\times TS \end{array}$$

where *M, N, K, W, H, and C* stand for *Feature Map Size in Conv Layers, Neuron Number in FC Layers, Kernel, Width, Height*, and *Number of Channels*; *Ops* is the number of required cycles to finish one computing operation [conditional branching: 1, ADD: 1, MUL: 3, and MAC: 4 based on edge deployment (Hennessy and Patterson, 2011)]; *bit* stands for needed bits of activation buffer between two NN layers in hardware. In SNN, the required operations are ADD and conditional branch (used to judge if the output spike should be fired). In the contrast, CNN needs more complicated MAC (MUL plus ADD operations). SNN processes single-bit activations while multi-bit (8b/16b/32b) activations are typically used in CNN implementations. To make a conservative evaluation of our design, we choose 8b for CNN in the calculation.

In the EEG experiment, our approach achieves an accuracy of 91.5% with a 96.1% reduction in computation complexity when the time step of spike-encoder is set 25. In the ECG experiment, with the same setting of time step, the proposed approach achieves an accuracy of 86.5% with an 80.5% reduction in computation complexity. In the EMG experiment, with the time step of 25, the proposed Spiking-CNN achieves an accuracy of 92.5% with an 80.7% reduction in computation complexity.

### 3.5. System implementation

**FIGURE 6:**
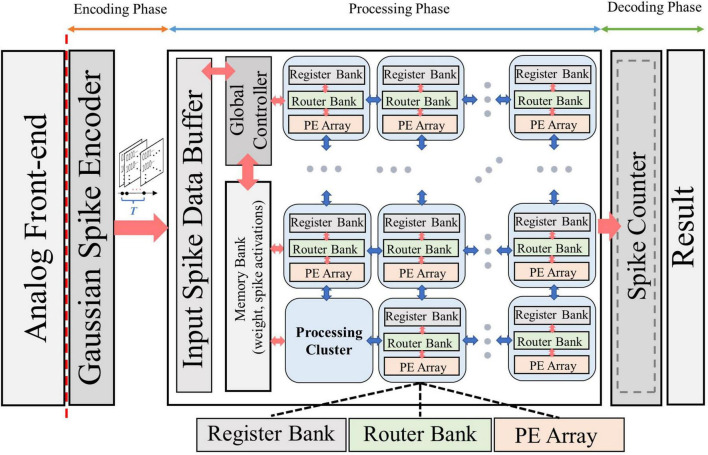
Overall system architecture design of NeuroCARE, consisting of encoding phase, processing phase and decoding phase.

**FIGURE 7:**
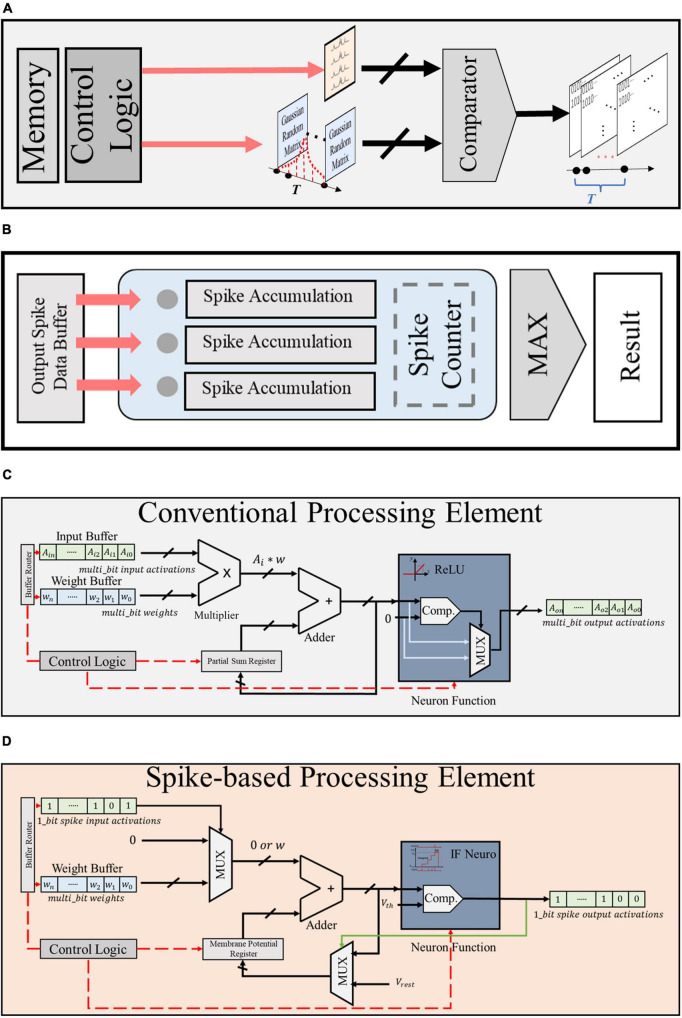
Module design of **(A)** Gaussian spike encoder, **(B)** output spike counter, **(C)** conventional CNN-based PE, and **(D)** proposed spike-based PE.

Figure 6 shows the baseline architecture used to implement the proposed framework. Various kinds of bio-signals are first encoded into spike sequences by the Gaussian spike encoder. The encoded spike data are then sent to the processing hardware and stored in the input spike data buffer. The architecture proposed in Eyeriss (Chen et al., 2017) is adopted in our work. It is optimized for CNN topology, hence suitable for the implementation of NeuroCARE. The output spike activations from the processing hardware are then sent to the spike counter to produce the result of classification. The proposed algorithm can be processed in the same way as demonstrated in Eyeriss (Chen et al., 2017). A global controller is used to control the data fetching and processing. All the processing is done in the processing cluster (PCL) arrays. Each PCL consists of a register bank, a router bank, and a processing element (PE) array. A memory bank is also connected with each PCL, which stores weights of the neural network and the spike activations between different network layers. Detailed designs of PEs are shown in Figure 7. Compared to other state-of-art works, our proposed method has two main advantages: (1) No multiplexer is required in the PCL of NeuroCARE, which greatly reduces the energy consumption in processing phase; (2) The bit resolution of activation data buffers in NeuroCARE is single-bit while conventional CNN-based implementations use multi-bit, thus our design requires much less energy consumption in data access.

### 3.6. Efficiency evaluations

To evaluate the implementation and energy efficiency of the proposed approach, key modules in NeuroCARE are designed and verified in the register-transfer level (RTL) level.

As presented in Figure 7A, the proposed Gaussian spike encoder is composed of a data buffer that stores the random Gaussian matrixes, a comparator and a control module. The controller fetches data from the buffer while the comparator compares the Gaussian value with the raw bio-signal to generate spike sequences. The proposed Gaussian spike encoder is practical with a very simple structure.

The design of spike counter is described in Figure 7B, which also has a simple structure. As mentioned in Part E of Section “3 Experiments and evaluation results,” the output spike activations are sent to the spike counter and stored in the output spike data buffer. When the whole processing procedures are completed through all the time steps, the total firing times of all the output neurons are calculated by the spike counter and the greatest one produces the result of classification.

Figures 7C, D present the detailed design of conventional CNN-based PE (CPE) and the proposed spike-based PE (SPE), respectively. As presented, the SPE has 1 adder, 2 mux, and 1 comparator, while the CPE requires 1 adder, 1 mux, 1 comparator, and 1 multiplexer. Compared to CPE, SPE shows advantages in two aspects: (1) the resolution of input and output buffers in SPE is single bit, thus brining reduction in memory assess and energy consumption; (2) the structure of SPE is much simpler and requires no multiplexer, thus further reduce the hardware cost and resource budget.

Evaluations on the energy consumption and area occupation of NeuroCARE system are conducted based on statistics from Horowitz (2014). The technology is assumed to be 45 nm and the voltage supply is assumed as 0.9 V. Thus, we get the comparison of energy consumption and area occupation between SPE and CPE under different data resolutions, which is shown in Table 2. The results demonstrate that using the same configuration, spike-based processing could achieve over 80% reduction in energy consumption and over 64.8% reduction in area occupation.

**TABLE 2:** Comparison of energy and area consumption between CNN-based PE (CPE) and spike-based PE (SPE).

Metrics	CPE	SPE
Technology	45 nm	
Voltage supply	0.9 V	
Weight data width	8b int / 32b int / 16b fp / 32b fp	8b int / 32b int / 16b fp / 32b fp
Activation data width	8b int / 32b int / 16b fp / 32b fp	**1 bit**
Energy per op.	0.25 pJ / 3.3 pJ / 2.0 pJ / 5.5 pJ	**0.03 pJ / 0.1 pJ / 0.4 pJ / 0.9 pJ**
Area	349 μm^2^ / 3,632 μm^2^ / 5,824 μm^2^ / 11,884 μm^2^	**36** μ**m^2^ / 137** μ**m^2^ / 1,360** μ**m^2^ / 4,184** μ**m^2^**
Energy reduction ration	**88% / 96.7% / 80% / 83.6%**	
Area reduction ratio	**89% / 96.2% / 77% / 64.8%**	
Efficiency acceleration	**7.58× / 79.7× / 2.17× / 1.79×**	

Energy reduction ration (ER) = 1−SPE energy / CPE energy. Area reduction ration (AR) = 1−SPE area / CPE area. Efficiency acceleration (EA) = 1 / [time step * (1−ER) * (1−AR)]. Bold values represent the advantages of the proposed method.

### 3.7. Overall evaluation

To evaluate the overall performance with considerations of both classification performance and resource expenses, we define a figure-of-merit (FOM) in this work as given in Eq. (20)


(20) $$\begin{array}{c} F\,O\,M=\frac{O\,v\,e\,r\,a\,l\,l\,S\,c\,o\,r\,e*E\,A\,f\,a\,c\,t\,o\,r}{\left(M\,U\,L+A\,D\,D+M\,E\,M\right)} \end{array}$$

According to Table 2, the efficiency acceleration (EA) factor ranges in 1.79 ∼ 79.7. To make a conservative evaluation of our design, we choose this scaling factor as 1.79.

The comparison between achieved results in this work and prior-art publications on metrics mentioned above is shown in Table 3. As reported, the proposed NeuroCARE shows advantages in both overall performance and energy efficiency compared to reported works. This approach stands as a channel-wise method without utilizing any extra feature extraction techniques, achieving high overall scores evaluated on three benchmarks. It also requires no MUL operations and little occupied memory, which makes it energy efficient. Therefore, NeuroCARE manages to achieve the highest FOM compared among all the other works.

**TABLE 3:** Comparison of performance with other works.

Classification task type	References	Model type	Dataset	No. of classes	Data type	Network depth	Channel wise	Overall score (%)	Accuracy (%)	F1 score	Number of ADD	Number of MUL	Memory occupied	FOM (%/M)
EEG based epileptic seizure prediction	NN2018 (Truong et al., 2018a)	STFT-CNN	MIT-CHB	2 class	Pre-processed	9 layers	No	91.7	81.2	81.4	1.17M	1.45M	3.81M	11.6
	BIBM2018 (Eberlein et al., 2018)	Wavelet-CNN	iEEG data	2 class	Pre-processed	21 layers	No	74.5	77.0	73.7	1.74M	2.20M	1.70M	16.26
	JBHI2020 (Zhang et al., 2020)	CSP-CNN	MIT-CHB	2 class	Pre-processed	7 layers	No	90.0	90.0	91.0	1.43M	1.97M	2.27M	15.9
	ISCAS2020 (Zhao et al., 2020)	BSD-CNN	MIT-CHB	2 class	Raw data	13 layers	Yes	94.33	97.0	94.69	5.54M	0.61M	0.067M	15.18
	This work	CNN	MIT-CHB	2 class	Raw data	13 layers	Yes	95.2	93.3	97.0	2.39M	2.84M	0.33M	17.12
		Spiking-CNN*						92.7	91.4	95.1	2.39M	0		61.0
ECG based arrhythmia detection	TBME2016 (Kiranyaz et al., 2016)	CNN	MIT-BIH (20 records)	5 class	Raw data	5 layers	No	87.6	98.3	76.4	0.25M	0.34M	0.64M	71.2
	ICHI2018 (Kachuee et al., 2018)	Residual CNN	PTB	5 class	Pre-processed	19 layers	No	95.4	95.9	95.1	4.83M	5.03M	1.33M	8.5
	BDAI2018 (Zhang et al., 2018)	LSTM	MIT-BIH	5 class	Pre-processed	12 layers	No	97.6	97.7	97.6	3.05M	2.71M	1.29M	13.8
	This work	CNN	MIT-BIH	4 class	Raw Data	10 layers	Yes	98.4	98.9	97.4	0.42M	0.61M	0.26M	77.1
		Spiking-CNN*						96.7	86.5	98.2	0.42M	0		254.5
EMG based Hand Gesture Recognition	IJCAI2017 (Du et al., 2017)	3D-CNN	Nina Pro	52 class	Pre-processed	8 layers	No	79.5	79.4	79.6	0.528M	0.530M	4.8M	13.6
	Sci. Rep.2016 (Geng et al., 2016)	Filter + CNN	Nina Pro	52 class	Pre-processed	9 layers	No	92.5	96.7	88.9	0.522M	0.524M	2.4M	18.9
	Frontiers2016 (Atzori et al., 2016)	Filter + CNN	Nina Pro	52 class	Pre-processed	7 layers	No	66.6	75.32	58.0	4.3M	4.3M	0.091	7.7
	This work	CNN	Nina Pro	3 class	Raw data	10 layers	Yes	95.5	96.1	94.9	0.57M	0.78M	0.51M	51.3
		Spiking-CNN*						85.7	92.5	82.7	0.57M	0		142.0

*The TS for spiking-CNN is set as 25 for comparison.

## 4. Conclusion

In this study, we propose NeuroCARE, a generic edge neuromorphic framework for healthcare applications. Modules include Gaussian spike encoder, neuromorphic neuron model, processing strategies, and the adaptive weight mapping method. To achieve high performance and reduce the required hardware resources, we first implemented CNN topologies, then scale and map the trained weights on Spiking-CNN correspondingly. Raw biomedical signals are processed in neuromorphic fashion on the time domain which significantly reduces the required memory and computation complexity.

As demonstrated by the implementation results, NeuroCARE achieves state-of-the-art classification results in epileptic seizure prediction, arrhythmia detection, and hand gesture recognition tasks, while managing to reduce the computation complexity by over 80.7%, reduce the computation energy consumption by over 80%, and reduce the area occupation by over 64.8% compared to CNN based methods, which greatly empowers the ultra-efficient neuromorphic intelligence for edge biomedical applications.

## Data availability statement

Publicly available datasets were analyzed in this study. This data can be found here: The EEG dataset for this study can be found in the CHB-MIT Dataset https://physionet.org/content/chbmit/1.0.0/. The ECG dataset for this study can be found in the MIT-BIH Arrhythmia Database https://physionet.org/content/mitdb/1.0.0/. The EMG dataset for this study can be found in the Ninapro DB1 Dataset http://ninaweb.hevs.ch/node/3.

## Author contributions

FT and JY designed the architecture and key modules of NeuroCARE. FT conducted all the relevant experiments and implementations. SZ preprocessed the dataset. MS supervised the study. All authors discussed the results, wrote the manuscript, and approved the submitted version.
